# Unilateral biportal endoscopic versus microscopic transforaminal lumbar interbody fusion for degenerative lumbar spinal stenosis in China: study protocol for a prospective, randomised, controlled, non-inferiority trial

**DOI:** 10.1136/bmjopen-2023-083786

**Published:** 2024-09-25

**Authors:** Zizhao Wu, Ting Luo, Yang Yang, Mao Pang, Ruiqiang Chen, Peigen Xie, Bu Yang, Lei He, Zifang Huang, Shangfu Li, Jianwen Dong, Bin Liu, Limin Rong, Liangming Zhang

**Affiliations:** 1 Third Affiliated Hospital of Sun Yat-Sen University, Guangzhou, Guangdong, China; 2 Department of Spine Surgery, Third Affiliated Hospital of Sun Yat-Sen University, Guangzhou, Guangdong, China; 3 Spine Surgery, Third Affiliated Hospital of Sun Yat-Sen University, Guangzhou, China

**Keywords:** Spine, Orthopaedic & trauma surgery, Endoscopic surgery, Minimally invasive surgery

## Abstract

**Introduction:**

Degenerative lumbar spinal stenosis is a common cause of low back or leg pain and disability in the elderly population. Patients with spinal stenosis who fail to respond to conservative treatment often require surgical interventions. Minimally invasive transforaminal lumbar interbody fusion (TLIF) with microscopic tubular technique (MT-TLIF) is a well-established procedure for lumbar spinal stenosis. Recently, a novel MIS technique, unilateral biportal endoscopic TLIF (UBE-TLIF), has been frequently performed to treat spinal stenosis. However, the efficacy and safety of using UBE-TLIF in this population have not been well examined.

**Methods and analysis:**

A total of 96 patients with lumbar spinal stenosis will be randomly assigned to the UBE-TLIF group or the MT-TLIF group at a 1:1 ratio to receive UBE-TLIF or MT-TLIF treatment respectively. The primary outcome is the Oswestry Disability Index (ODI) score at 1 year after receiving the surgery. Secondary outcomes include the ODI scores at additional time points, Visual Analogue Scale score, 36-Item Short Form Survey questionnaire, EuroQol 5 Dimensions questionnaire, radiological measurements (disc height, lumbar lordosis angles and vertebral fusion rate) and general condition during hospitalisation.

**Ethics and dissemination:**

This protocol is approved by the Medical Ethics Committee of the Third Affiliated Hospital of Sun Yat-sen University. All participants of the study will be well informed and written informed consent will be requested. Findings from this trial will be published in peer-reviewed publications, specifically in orthopedic and spinal journals. The completion of this study will not only examine the use of UBE-TLIF in lumbar spinal stenosis but also provide helpful clinical references.

**Trial registration number:**

ChiCTR2300069333.

**STRENGTHS AND LIMITATIONS OF THIS STUDY**

- This is a prospective, randomised controlled, non-inferiority clinical study to compare the surgical procedure of unilateral biportal endoscopic transforaminal lumbar interbody fusion (TLIF) with microscopic tubular technique TLIF for the treatment of degenerative lumbar spinal stenosis in terms of the clinical safety and efficacy.
- All surgeries are performed by surgeons who have 10-year operation experience and are familiar with the two surgical procedures.
- The data are collected from a single institution with a relatively small sample size.
- The surgeons cannot be blinded for the study because the intervention is surgery and the protocols for two surgeries are different.

## Introduction

 Degenerative lumbar spinal stenosis (DLSS) can cause a series of symptoms, such as low back and leg pain as well as intermittent claudication. Patients who fail to respond to conservative treatment often require surgery interventions.^1^ It is generally agreed that for patients who have severe spinal stenosis and (or) spondylolisthesis, sufficient neural decompression and lumbar interbody fusion have been demonstrated to be an effective approach to improve clinical outcomes.^2 3^ However, traditional open surgery is associated with a series of drawbacks such as abundant blood loss, significant damage to soft tissue and long hospital stay.^4 5^

Anand *et al* first reported the microscope-assisted transforaminal lumbar interbody fusion (TLIF) technique which has the advantages of smaller incision, less soft tissue damage, decreased blood loss and enhanced postoperative recovery compared with the open surgery.^6^,^8^ With the development of surgical technique and instrument, the biportal endoscopic spinal fusion was first implemented in 2012 by Osman *et al* and Dong Hwa Heo *et al* first used minimally invasive TLIF with biportal endoscopic in 2017.^9 10^ Previous studies show that unilateral biportal endoscopic TLIF (UBE-TLIF) has less blood loss, reduced postoperative pain and faster postoperative recovery compared with conventional methods.^11 12^ Moreover, due to the high-definition field of view, the separation of the working and the observation channel in UBE-TLIF is relatively more convenient.^13^ However, it has the disadvantages such as relatively deep learning curve, limited indications and long operation time, which may increase perioperative complications.^14^ Therefore, its efficacy is uncertain, and there is still no high-level evidence to support whether UBE-TLIF is non-inferior to microscopic tubular technique (MT-TLIF) in terms of clinical outcomes up to now.

Here, a prospective, randomised controlled, non-inferiority clinical study was designed to compare the surgical procedure of UBE-TLIF with MT-TLIF for the treatment of DLSS in terms of the clinical safety and efficacy in detail.

### Research aim

The purpose of this study is to test whether UBE-TLIF is non-inferior to MT-TLIF in treating patients with DLSS in terms of efficacy and safety.

## Methods and analysis

### Patient and public involvement

There is no patient and/or public involvement in the overall design, conduct, reporting or dissemination of this study. The expected study duration is from February 2023 to March 2025.

### Study design and settings

**Figure 1:**
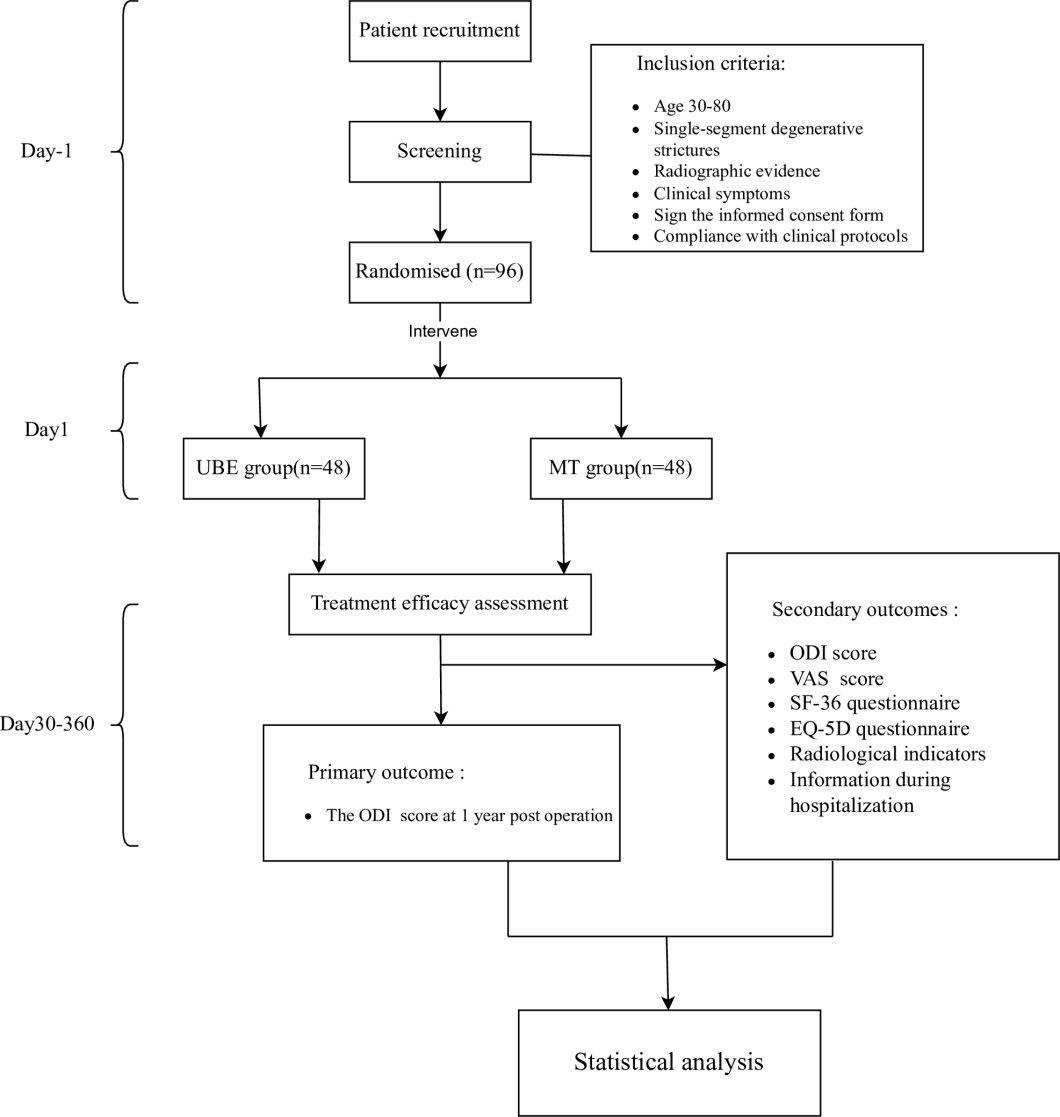
Study flow chart. EQ-5D, EuroQol 5 Dimensions; ODI, Oswestry Disability Index; SF-36, 36-Item Short Form Survey; UBE, unilateral biportal endoscopic; VAS, Visual Analogue Scale.

This is a prospective, randomised, controlled, non-inferiority clinical study. A total of 96 eligible patients will be randomly assigned to the MT-TLIF arm or UBE-TLIF arm at a 1:1 ratio and receive treatments at the Third Affiliated Hospital of Sun Yat-sen University. A diagram of the trial design is shown in figure 1. The primary outcome is the Oswestry Disability Index (ODI) score at 1-year postoperation. The study has been registered and can be accessed online on the China Clinical Trial Registration Center website (https://www.chictr.org.cn/), with the registration date being 14 March 2023. Trial registration data of this study is in online supplemental table 1.

### Patient selection

A trained research nurse will introduce the trial to the subjects and have a discussion with them. Written consent will be obtained from subjects who are willing to participate in the trial. This study will include patients aged between 30 and 80 years with the lower extremity pain and/or neurogenic intermittent claudication and confirmed by MRI as single-level DLSS. X-ray confirmed degenerative or isthmic spondylolisthesis ≤grade II in Meyerding grade, or lumbar spine instability, or decrease in disc height (DH) accompanied by intervertebral foraminal stenosis. Moreover, these symptoms persist for at least 3 months before the surgery. The inclusion and exclusion criteria are presented in table 1.

**Table 1:** Inclusion and exclusion criteria

Inclusion criteria	Exclusion criteria
Age 30–80 years old	Highly degenerative and isthmus bifida spondylolisthesis (Meyerding grade III and IV)
Lower extremity pain and/or neurogenic intermittent claudication, and confirmed by MRI as single-level degenerative lumbar spinal stenosis	Narrowing or slipping of more than one segment that requires decompression or fusion of more than two segments
Degenerative or isthmus fissure ≤Meyerding grade II, or lumbar vertebra instability (forward or posterior sagittal displacement ≥3 mm or ≥8%, or sagittal angle change ≥15°) or decrease in DH accompanied by intervertebral foraminal stenosis	Presence of any other neurological disease or vascular disease of the legs that may interfere with the study assessment
Symptoms lasting at least 3 months before surgery	Any segment fusion surgery of the lumbar vertebra
Sign the informed consent form, be able to understand the informed consent form and follow-up related questionnaires	Other spinal diseases (such as spinal infection, ankylosing spondylitis, other inflammatory spondylitis, spinal tumours, spinal trauma)
Agreed to comply with clinical protocols and willing to adhere to follow-up schedules and requirements	Failure to perform baseline and/or follow-up MRI or X-ray as required
	Cognitive and psychological disorders (such as Alzheimer’s disease, intellectual disability or substance abuse)
	Other diseases deemed inappropriate to participate in the study by the surgeons

DHdisc height

### Randomisation

A randomised block design will be adopted with admission time being the matching factor. Specifically, four adjacent patients who are admitted in the same month will be considered as a block. Within each block, the four subjects can be arranged in six distinct permutations: AABB, ABAB, ABBA, BAAB, BABA and BBAA, corresponding to the random numbers 1–6, respectively. These permutations are then randomly assigned to two groups, designated as group A and B. Following the determination of the arrangements, the subsequent phase entails a random selection from the six pre-established sequences. For each selection, a number is randomly drawn from the set ranging from 1 to 6. For example, the selection of the number 5 is specifically designated to represent the ‘BABA’ arrangement. The four subjects who enter this block are assigned to groups B, A, B and A, respectively. Random numbers ranging from 1 to 6 will be generated by a research assistant, who is blinded to the trial, using a personal computer prior to the commencement of patient recruitment. Subsequently, the random numbers will be concealed in sequentially coded, opaque and sealed envelopes and opened by another research assistant.

### Blinding

Since the wounds on the body surface are basically the same size after surgery, the subjects do not know their specific surgical methods. The clinical assessment process, including baseline levels and self-report questionnaires, will be performed by clinicians who are unaware of subject group assignments. Evaluation data will be stored in electronic medical records and collected in parts according to the case report form (CRF). Statistics can only be carried out after all data are collected. All statistical raw data do not provide grouping information. The main researcher will provide specific information on grouping after all information has been entered.

### Baseline data

Baseline data including demographics, comorbidity, ODI score, Visual Analogue Scale (VAS) score, 36-Item Short Form Survey (SF-36) and EuroQol 5 Dimensions (EQ-5D) questionnaire will be collected. X-rays that measure the DH of operated level and lumbar lordosis (LL) angles will be conducted. DH can be calculated as the average value of the height from anterior part, middle part and posterior part in the same disc. LL angle is defined as the angle between the upper endplate of vertebra Lumbar one and Sacral 1.^15^,^17^

### Surgical procedure

To avoid technical bias, all surgeries are performed by surgeons who have at least 10 years surgical experience and are familiar with the two surgical procedures (more than 100 cases of experience).

For UBE-TLIF,^18^ observation and operation channels are established independently without intrainterfering. The endoscope used in this operation will be the same one that used for the knee arthroscope. After attaching the spinal endoscope, the rongeur removes part of the lamina, followed by ligamentum flavum resection and further nerve decompression. Then the annulus fibrosus will be separated, and the degenerated intervertebral disc tissue will be removed to process the cartilage endplate. The removed autologous bone will be attached to a allograft bone before inserting it into a suitable cage which further to be implanted into the intervertebral space through the operation channel. Finally, percutaneous pedicle screws will be inserted and fixed.

For MT-TLIF,^18^ a working channel is established within the intermuscular space of the multifidus muscle. Specifically, a 3.5–4.5 cm longitudinal incision will be made. Then, a series of dilators ranging from small to large are inserted and a suitable tubular retractor is placed. With the aid of microscope, part of the articular processes, laminae and corresponding ligamentum flavum will be removed to decompress the nerve root and dural sac. Then the intervertebral disc will be removed to process the cartilage endplate. A single fusion cage with autologous and allogeneic bone will be inserted before placing percutaneous pedicle screws and fixation rods.

### Outcome measurements

#### Primary outcome

The main outcome of the study is the ODI score at 1-year postoperation, which is a 10-item questionnaire specific to low back pain patients, including pain intensity, personal care (washing, dressing etc), lifting, walking, sitting, standing, sleeping, sex life (if applicable), social life and travelling. Each of the 10 items is scored from 0 to 5. Total scores are calculated by dividing the actual by the maximum score of 50 and converting it to a percentage. The score of 100% is recognised as the highest score, signifying the greatest dysfunction severity.^19 20^

The minimal clinically important difference (MCID) is a threshold value that patients and clinicians perceive as clinically meaningful. The clinically acceptable MCID in reported ODI scores is 8–12. However, to date, there is no consensus on calculating the optimal MCID for the ODI scores in groups.^21^,^23^ Consequently, following discussion, we adopted 8 as the MCID within clinically acceptable range. By comparing the ODI score 1 year after surgery, we can determine whether UBE-TLIF is not inferior to MT-TLIF within the non-inferiority margin of 8.

Considering that the condition of most enrolled subjects will be stabilised at 1-year postoperation, the assessment time frame of 1-year ODI is between 12 and 15 months after the operation. In case subjects do not return to the hospital for follow-up on time during this period, a telephone follow-up will be conducted to complete the ODI questionnaire.

#### Secondary outcome

The secondary outcomes of this study will assess ODI score at additional time points, VAS, SF-36, EQ-5D, radiological indicator (DH, LL angles and vertebral fusion rate) and hospitalisation information. The data collection schedule is presented in table 2. For those who cannot come for follow-ups, our research assistant will contact them by phone or email to complete the questionnaire.

**Table 2:** The time schedule for collection of data for this trial

	Day≤−1	Day1	Day2–29	Day30±7 days	Day90±7 days	Day180±7 days	Day360±7 days
Inclusion and exclusion criteria	**√**						
Signing of informed consent form	**√**						
Demographic assessment	**√**						
Physical examination	**√**						
Operation time/Intraoperative blood loss		**√**					
Serum CK/CRP			**√**				
Length of stay			**√**				
ODI score	**√**			**√**	**√**	**√**	**√**
VAS score	**√**			**√**	**√**	**√**	**√**
SF-36-PF	**√**			**√**	**√**	**√**	**√**
SF-36-BP	**√**			**√**	**√**	**√**	**√**
QoL EQ-5D	**√**			**√**	**√**	**√**	**√**
Disc height	**√**				**√**	**√**	**√**
Lumbar lordosis angles	√				√	√	√
Vertebral fusion rate							**√**
Complications		**√**	**√**	**√**	**√**	**√**	**√**
Adverse events		**√**	**√**	**√**	**√**	**√**	**√**

√, This refers to the data collection at the scheduled time.

BPbody painCKcreatine phosphokinaseCRPC reactive proteinEQ-5DEuroQol 5 DimensionsODIOswestry Disability IndexPFphysical functioningQoLquality of lifeSF-3636-Item Short Form SurveyVASVisual Analogue Scale

#### ODI score

The ODI scores were assessed at 1 month, 3 months and 6 months postoperatively. Scores are determined following the aforementioned method.

#### VAS score

The VAS score records an individual’s self-rated health status on a vertical VAS, which was assessed at 1 month, 3 months, 6 months and 12 months postoperatively. A 10 cm ruler with 10 gradations marks the pain scale, with 0 for no pain and 10 for the worst, unbearable pain. Higher scores equate to more severe pain.^24^

#### SF-36 questionnaire

SF-36 questionnaire is used to evaluate health-related quality of life. The evaluation covers eight specific domains: physical functioning (PF), role-physical, body pain (BP), general health, vitality, social functioning, role-emotional and mental health. The main statistical indicator of SF-36 is to calculate the health score of eight dimensions. The initial score of each dimension is equal to the sum of the scores of all items within each, higher score on the SF-36 reflects superior health status.^25^ In this study, we focus on two aspects: PF and BP, conducted at 1 month, 3 months, 6 months and 12 months postoperatively.

#### EQ-5D questionnaire

The EQ-5D health scale features a descriptive system and EQ-VAS. The descriptive system encompasses five dimensions, including mobility, self-care, usual activities, pain/discomfort and anxiety/depression. Each of the five items is scored from 1 to 5. The EQ-VAS, a 20 cm vertical scale, ranges from 0, indicative of the poorest health state, to 100, reflective of optimal health.^26^ Participants are required to pinpoint their health status on this scale, offering a snapshot of their well-being on the survey day at 1 month, 3 months, 6 months and 12 months postoperatively.

#### Radiological indicators

DH and LL angles are measured by X-rays. The reduction in DH correlates with the progression of intervertebral disc degeneration, characterised notably by intervertebral space stenosis. Thus, the magnitude of DH reduction serves as an indicator of the degenerative process’s severity. LL angles over 10° suggest progression risk in spondylolisthesis. The approach to measurement is meticulously detailed in the prior section. An experienced spine physician and radiologist with ten years of experience will simultaneously evaluate the intervertebral fusion of the subjects based on CT scans. The fusion criteria are shown in table 3.^27^

**Table 3:** Bridwell intervertebral bone graft fusion evaluation system

Grade	Description
I	Fused with remodelling and trabeculae present
II	Graft intact, not fully remodelled and incorporated, but no lucency present
III	Graft intact, potential lucency present at top and bottom of graft
IV	Fusion absent with collapse or resorption of graft

#### Information during hospitalisation

Information during the hospitalisation includes operation time, hospital stay, intraoperative blood loss, serum creatine phosphokinase (CK) and C reactive protein (CRP) level. Serum levels of CK and CRP will be collected within 2 days after receiving the surgery, which serves as a crucial measure to delineate the extent of soft tissue trauma experienced during the surgical procedure

### Data collection, management and monitoring

The data safety monitoring committee (DSMC) will be composed of two expert spine surgeons and an expert statistician. Efficacy and safety data for all patients will be reviewed throughout the study. A standard data collection and management system including CRF and electronic data capture will be adopted. CRF data filling and management will be carried out by research assistants who are blinded from the study. All participant identities and recorded data will be kept confidential, and only the principal investigator and DSMC will have access to the data for this study. The names of subjects will not be identified in any publications from this study.

According to the data collection schedule (table 1), data will be collected before surgery, on 2 days, in 1 month, 3 months, 6 months and 12 months after the surgery.

### Statistical analysis

In this study, three analysis sets will be required. For the safety analysis set, any adverse event (AE) will be recorded from patients who have undergone UBE-TLIF or MT-TLIF following randomisation. In the full analysis set, all participants who were randomised to the surgical intervention and for whom complete data sets were accessible after randomisation. In instances of missing data, we employed the last observation carried forward and baseline observation carried forward methods for imputation. For the per-protocol cohort, at least 90% of subjects should fulfil the following criteria: (1) receive either UBE-TLIF or MT-TLIF treatments; (2) with no violation of the protocol; (3) complete the full follow-up and (4) obtain all required data. Continuous variables, including VAS score, ODI score, operation time, intraoperative blood loss, serum CK and CRP level, DH, LL, SF-36, QoL EQ-5D as well as hospital stay, will be expressed as means±SDs, while categorical data, including vertebral fusion rate, will be shown as frequencies. The independent t-test and Pearson χ^2^ will be applied to analyse continuous and categorical variables, respectively. If indicated, statistical analysis between subgroups will be performed. All statistical analyses will be performed using Statistical Product and Service Solutions (V.26.0, IBM) by the principal biostatistician. A p<0.05 (two sided) will be considered statistically significant.

### Sample size

The MCID for ODI scores reported in similar studies is 8–12.^22 23^ After discussing among experienced professors from the spine surgery department, 8 in ODI score is chosen as the margin of non-inferiority (ðN) in this study. Based on the Consolidated Standards of Reporting Trials 2010 statement,^28^ it is assumed that the expected response is the same in both groups, the estimated true difference between the two surgeries is 0, and the ðN is 8. The SD is estimated to be 13 based on previous studies.^29^ The test level is one sided (α=0.025), and the test power (1−β) is 0.8. Considering that 10% of population may loss to follow-up, a total of 96 subjects are required in this clinical study.

### AE and serious AE

Surgery-related AEs from both arms will be reported and documented throughout the study period.

Intraoperative complications include dural tear, nerve root injury, pedicle screw misplacement, endplate fracture, cardiopulmonary complications, allergic reactions and other unpredictable complications. Postoperative complications include cerebrospinal fluid leakage, neurological deficit or deterioration, excessive postoperative drainage volume (>400 mL/day), postoperative infection, surgery-related thrombotic events, pedicle screw breakage, cage subsidence or migration, cardiopulmonary complications, haematoma within the spinal canal and other unpredictable complications.^30^,^32^

During the intervention and follow-up periods of this trial, any AE will be identified promptly and managed properly.

## Discussion

The current protocol describes the rationale, non-inferiority design and methods of this randomised, controlled clinical trial that compares the efficacy and safety of UBE-TLIF to MT-TLIF in patients of DLSS. The non-inferiority design can not only demonstrate the efficacy of UBE-TLIF compared with MT-TLIF but also focus on exploring the advantages beyond efficacy. Furthermore, it can avoid ethical concerns and has more ethical value than placebo-controlled or no-treatment-controlled clinical trials.^33^ Moreover, to avoid potential bias, all surgeries are performed by surgeons who have 10-year operation experience and are familiar with the two surgical procedures.

Moreover, the primary outcome in this study is the ODI score at 1-year postoperation. MCID was used to determine the sample size of this trial, which may facilitate the final analysis. Additionally, a majority of the outcomes in this study are self-reported by the subjects, including the VAS score, ODI score, SF-36 questionnaire and EQ-5D questionnaire, which will help minimise observation bias to some extent.

However, there are still some potential limitations that need to be addressed. The surgeon cannot be blinded for the study because the protocols for the two surgeries are different. However, to reduce bias as much as possible, investigators who are blinded in the group assignment process will perform the clinical assessment, including baseline evaluation. Additionally, all data will be collected from a single institution with a relatively small patient number. Larger multicentre studies are needed to confirm the efficacy and safety of MT-TLIF and UBE-TLIF in treating DLSS in future.

In conclusion, this study aims to compare the efficacy and safety of MT-TLIF and UBE-TLIF in treating patients with DLSS. The completion of this study will not only examine the use of UBE-TLIF in lumbar spinal stenosis but also provide helpful clinical references.

## Ethics and dissemination

This study will follow the ethical guidelines of the Declaration of Helsinki of the World Medical Assembly and relevant norms as well as regulations of clinical research. The study will begin only after obtaining ethics committee approval. Before the start of the study, researcher will inform the subjects of all relevant contents of the clinical study in an easy-to-understand language, and their right to withdraw from the study at any time. Only patients who signed the informed consent will be enrolled in the study.

## supplementary material

10.1136/bmjopen-2023-083786 online supplemental file 1
