# Supplementary material for: Unilateral biportal endoscopic versus microscopic transforaminal lumbar interbody fusion for degenerative lumbar spinal stenosis in China: study protocol for a prospective, randomised, controlled, non-inferiority trial
Source: BMJ Open. 2024 Sep 25;14(9):e083786. doi: 10.1136/bmjopen-2023-083786 (PMC11425936; doi:10.1136/bmjopen-2023-083786)
Supplement: online supplemental file 1 [file bmjopen-14-9-s001.pdf]

Table S1. Trial registration data of this study

Data category	Information
Registration number	ChiCTR2300069333
Date of Registration	14 March, 2023
Primary sponsor	The Third Affiliated Hospital of Sun Yat-sen University
Primary sponsor's address	600 Tianhe Road, Tianhe District, Guangzhou, Guangdong
Source(s) of funding	"Voyage Program" grant number YHJH202203 and "The Five Significant Programs" grant number WW201903 of the Third Affiliated Hospital of Sun Yat-sen University.
Study leader	Zhang Liangmin [zhanglm36@mail.sysu.edu.cn]
Scientific title	A prospective, randomized controlled, non-inferior clinical study: comparison of the efficacy of TLIF in lumbar spinal stenosis under unilateral biportal endoscopic technique and microscope-assisted technique
Countries of recruitment	China, Third Affiliated Hospital of Sun Yat-sen University
Target disease	Degenerative lumbar spinal stenosis
Study type	Interventional study
Inclusion and exclusion criteria	Inclusion criteria: Age 30-80 years old, single-level degenerative lumbar spinal stenosis<br>Exclusion criteria: Highly degenerative and isthmus bifida spondylolisthesis, narrowing or slipping of more than one segment, Presence of any other neurological disease or vascular disease of the legs, any segment fusion surgery of the lumbar vertebra, other spinal diseases, cognitive and psychological disorders.
Interventions	Group A: Minimally invasive transforaminal lumbar interbody fusion with microscopic tubular technique (MT-TLIF)<br>Group B: Minimally invasive transforaminal lumbar interbody fusion with unilateral biportal endoscopic (UBE-TLIF)
Primary outcome(s)	Oswestry Disability Index (ODI) score at 1 year after receiving the surgery
Secondary outcome(s)	ODI scores at additional time points, Visual Analogue Scale (VAS) score, 36-Item Short Form Survey (SF-36) questionnaire, EuroQol Five Dimensions (EQ-5D) questionnaire, disc height (DH), lumbar lordosis (LL) angles, vertebral fusion rate, and general condition during hospitalization
Sample size	96
Recruiting status	Recruiting
