# Supplementary material for: Unilateral biportal endoscopic versus microscopic transforaminal lumbar interbody fusion for degenerative lumbar spinal stenosis in China: study protocol for a prospective, randomised, controlled, non-inferiority trial
Source: BMJ Open. 2024 Sep 25;14(9):e083786. doi: 10.1136/bmjopen-2023-083786 (PMC11425936; doi:10.1136/bmjopen-2023-083786)
Supplement: Supplementary file 2 [file bmjopen-2023-083786.reviewer_comments.pdf]

PEER REVIEW HISTORY

BMJ Open publishes all reviews undertaken for accepted manuscripts. Reviewers are asked to complete a checklist review form and are provided with free text boxes to elaborate on their assessment. These free text comments are reproduced below.

ARTICLE DETAILS

**TITLE (PROVISIONAL)**	Unilateral Biportal Endoscopic versus Microscopic Transforaminal Lumbar Interbody Fusion for Degenerative Lumbar Spinal Stenosis in China: study protocol for a prospective, randomized, controlled, non-inferiority trial
**AUTHORS**	Wu, Zizhao; Luo, Ting; Yang, Yang; Pang, Mao; Chen, Ruiqiang; Xie, Peigen; Yang, Bu; He, Lei; Huang, Zifang; Li, Shangfu; Dong, Jianwen; Liu, Bin; Rong, Limin; Zhang, Liangmin

VERSION 1 - REVIEW

**REVIEWER NAME**	Gilmore, Sarah
**REVIEWER AFFILIATION**	St Vincent's Private Hospital, Physiotherapy Department
**REVIEWER CONFLICT OF INTEREST**	Nil
**DATE REVIEW RETURNED**	12-Feb-2024

**GENERAL COMMENTS**	Thank you for the opportunity to review this manuscript - overall it appears to be a well designed study that will provide important information on the efficacy and safety of spinal surgery techniques.<br><br>- While generally being well written, some word/phrases need to be reviewed to ensure they are appropriate, for example, the use of "refractory" in the second sentence of the introduction, the patient selection paragraph needs to be checked for grammar, and the paper needs to be reviewed to ensure the tense is consistent the whole way through.<br>- It would be useful to expand on what you mean by efficacy - I can see that function is your primary outcome and you are also measuring pain, QOL, and radiological data, it would be good to have this summarized early in the paper and to include why you are measuring these outcomes.<br>- Please provide more information about the outcome measures - why you chose them, how they are scored and their validity/reliability in this patient population.

**REVIEWER NAME**	Minetama, Masakazu
**REVIEWER AFFILIATION**	Wakayama Medical University Kihoku Hospital, Spine Care Center
**REVIEWER CONFLICT OF INTEREST**	I have no competing interests.
**DATE REVIEW RETURNED**	21-May-2024

GENERAL COMMENTS	Thank you for the opportunity to review this study protocol of unilateral biportal endoscopic versus microscopic transforaminal lumbar interbody fusion for degenerative lumbar spinal stenosis. This reviewer has some questions, which should be addressed by authors prior to publication.<br><br>About the inclusion criteria, this reviewer wonders if patients aged 30 years are too younger for degenerative lumbar spinal stenosis.<br><br>About the randomization, how to generate random numbers, and who will open envelopes and assign patients are not described.<br><br>On p.36-37 “we can determine whether UBE-TLIF is not inferior to MT-TLIF within the non-inferiority margin of 8.”, it should be added the explanation for the basis of “margin of 8” and attached the reference.<br><br>About outcome measurements, it is unclear whether assessments will be blinded, because who will hand over questionnaires are not mentioned in the methods section.<br><br>On p. line 59- p. line 3 “subjects will be excluded if they were never evaluated after receiving the operation”, does this mean that they will be excluded from statistical analyses, and non-intention-to-treat analyses? If this study applies ITT analyses, it should not exclude the participants who were enrolled and performed baseline assessments.<br><br>On P.8, line 15 in the statistical analysis section, VAS and ODI scores are not categorical data. If you have planned to analyze successful rates of archiving MCID values for VAS and ODI, it should be mentioned in the protocol.<br><br>There is a discrepancy between the sample size calculation and plans of statistical analysis. The p value is set at 0.05 in the statistical section, but sample size is set at 0.025 in the sample size calculation. The sample size should be calculated based on statistical analyses in the study.<br><br>AEs in the abstract section, and UBE-TLIF and MT-TLIF on p.3, line 47 and 60 are required their full sentences.<br><br>On p.6, line 11 “Specifically, a 3.5-4.5cm longitudinal incision will be made.” duplicates next sentence, so it should be removed.

VERSION 1 – AUTHOR RESPONSE

Reviewer: 1

Special thanks to Dr. Sarah Gilmore for your good comments and suggestion. According to your helpful suggestions, we have revised the manuscript.

1.While generally being well written, some word/phrases need to be reviewed to ensure they are appropriate, for example, the use of "refractory" in the second sentence of the introduction, the patient selection paragraph needs to be checked for grammar, and the paper needs to be reviewed to ensure the tense is consistent the whole way through.

Answer: As suggested, we have revised the sentences in the introduction into “Patients who fail to respond to conservative treatment often require surgery interventions.” We have also corrected the grammar of the patient selection paragraph meticulously and revised the paper to ensure tense is consistent the whole way through.

2.It would be useful to expand on what you mean by efficacy - I can see that function is your primary outcome and you are also measuring pain, QOL, and radiological data, it would be good to have this summarized early in the paper and to include why you are measuring these outcomes.

Answer: According to your helpful suggestions, we have re-written the secondary outcome part. We have summarized these outcomes in the first paragraph and all the secondary outcomes we selected such as pain, QOL, and radiological data are listed point-by-point. We have provided additional details on these secondary outcomes, explaining their measurement, scoring, and validity in the population.

3.Please provide more information about the outcome measures - why you chose them, how they are scored and their validity/reliability in this patient population.

Answer: According to your helpful suggestions, we have re-written the secondary outcome part where we provided additional details on these secondary outcomes, explaining their measurement, scoring, and validity in the population.

Thanks again for your valuable suggestion. We hope the modifications would make you satisfied.

Reviewer: 2

We appreciate the constructive suggestions provided by Dr. Masakazu Minetama. We have modified the paper as you suggested.

1.About the inclusion criteria, this reviewer wonders if patients aged 30 years are too younger for degenerative lumbar spinal stenosis.

Answer: As you are concerned, if patients aged 30 years are too younger for degenerative lumbar spinal stenosis. According to our clinic experience, due to unhealthy lifestyle such as sedentary, LSS has exhibited an increasing prevalence among younger demographics. We have also reviewed the relevant literature on randomized controlled trials of DLSS. We found that Ho-Joong Kim et al 2016 enrolled subjects who range of 20 to 75 years, Yvonne Lindbäck et al 2018 from 25 to 80, Erland Hermansen et al 2022 over 18. Therefore, we feel that patients aged 30 years is justified. We hope this explanation addresses your concerns.

[PMID: 27045252; PMID: 29253630; PMID: 35344046]

2. About the randomization, how to generate random numbers, and who will open envelopes and assign patients are not described.

Answer: According to your helpful suggestions, we have re-written and added more details for the randomization part. Random numbers were generated by a research assistant using a personal computer, who was blinded to the trial. Subsequently, the random numbers in the envelopes were opened by another research assistant. According to the random numbers and our study design, participates were allocated to either group UBE-TLIF or MT-TLIF. We have thoroughly updated our paper to include a detailed exposition on the ways of random number generation and the methodology for subsequent group allocation based on these numbers.

3.On p.36-37 “we can determine whether UBE-TLIF is not inferior to MT-TLIF within the non-inferiority margin of 8.”, it should be added the explanation for the basis of “margin of 8” and attached the reference.

Answer: We have checked the literature carefully and added more references on Minimal Clinically Important Difference (MCID) and margin of 8 into the PRIMARY OUTCOME part. [PMID: 18201937; PMID: 27870805; PMID: 28327114] As the explanation we added “The clinically acceptable MCID in reported ODI scores is 8-12. However, to date, there is no consensus on calculating the optimal MCID for the ODI scores ingroups. Consequently, following the discussion, we adopted 8 as the MCID within clinically acceptable range.” in revised paper.

4.About outcome measurements, it is unclear whether assessments will be blinded, because who will hand over questionnaires are not mentioned in the methods section.

Answer: Based on your comments, we have provided more information about the assessment in the revised paper. “The clinical assessment process, including baseline levels and self-report questionnaires were performed by clinicians who were unaware of subject group assignments.” Participants, evaluators, and statisticians will be blinded in this study. We feel sorry for the omission of the blinding method in the initial manuscript and have now added a detailed description in the revised version.

5.On p. line 59- p. line 3 “subjects will be excluded if they were never evaluated after receiving the operation”, does this mean that they will be excluded from statistical analyses, and non-intention-to-treat analyses? If this study applies ITT analyses, it should not exclude the participants who were enrolled and performed baseline assessments.

Answer: We thank for the reviewers’ comment, and we agree with your opinion. Subjects should not be excluded if they were never evaluated after receiving the operation for the ITT analyses. Participants who deviated from the study protocol or withdrew from the study were still maintained within their initially randomized cohort for analytical purposes. In response to the comment, we have corrected the inaccurate expression and added a description of participants lost to follow-up. “In the full analysis set, all participates who were randomized to the surgical intervention and for whom complete data sets were accessible after randomization. In instances of missing data, we employed the Last Observation Carried Forward (LOCF) and Baseline Observation Carried Forward (BOCF) methods for imputation.”

6.On P.8, line 15 in the statistical analysis section, VAS and ODI scores are not categorical data. If you have planned to analyze successful rates of archiving MCID values for VAS and ODI, it should be mentioned in the protocol.

Answer: We feel sorry for our careless. Based on your comments, we have made the corrections that the VAS and ODI scores are continuous variables. Thank you again for your correction.

7.There is a discrepancy between the sample size calculation and plans of statistical analysis. The p value is set at 0.05 in the statistical section, but sample size is set at 0.025 in the sample size calculation. The sample size should be calculated based on statistical analyses in the study.

Answer: As you are concerned, the p value of the sample size calculation and statistical analyses should be consistent. In the sample size calculation, the test level is one-side ($\alpha=0.025$), and two-side are 0.5. Simultaneously, we have searched the literature carefully, these three articles follow the same approach as ours. The sample size was determined with alpha of 0.025 (one-side), and $p < 0.05$ (two-side) was considered statistically significant. (PMID: 36339599; PMID: 34539392; PMID: 34760706). Since we did not express 0.05 is two-side clearly, we have modified it into “$P < 0.05$ (two-side) will be considered statistically significant”.

8.AEs in the abstract section, and UBE-TLIF and MT-TLIF on p.3, line 47 and 60 are required their full sentences.

Answer: We feel sorry for our care mistakes. In our resubmitted manuscript, we have provided the full sentences of adverse events (AEs), unilateral biportal endoscopic transforaminal lumbar interbody fusion (UBE-TLIF), and microscopic tubular technique transforaminal lumbar interbody fusion (MT-TLIF).

9.On p.6, line 11 “Specifically, a 3.5-4.5cm longitudinal incision will be made.” duplicates next sentence, so it should be removed.

Answer: We feel sorry for our care mistakes. In our resubmitted manuscript, we have removed the duplicated sentence.

Thank you again for taking the time to review this paper a second time. We sincerely hope that the revise can meet your requirement.

VERSION 2 - REVIEW

**REVIEWER NAME**	Minetama, Masakazu
**REVIEWER AFFILIATION**	Wakayama Medical University Kihoku Hospital, Spine Care Center
**REVIEWER CONFLICT OF INTEREST**	I have no competing interests.
**DATE REVIEW RETURNED**	03-Aug-2024

**GENERAL COMMENTS**	I do believe that the authors have addressed the issues that I raised and that the manuscript is significantly better than before. This is a nice paper and I would recommend publication.
